# Functioning Endocrine Outcome after Endoscopic Endonasal Transsellar Approach for Pituitary Neuroendocrine Tumors

**DOI:** 10.3390/jcm12082986

**Published:** 2023-04-20

**Authors:** Gabriele Molteni, Nicole Caiazza, Gianfranco Fulco, Andrea Sacchetto, Antonio Gulino, Daniele Marchioni

**Affiliations:** Division of Otorhinolaryngology, Department of Surgery, Dentistry, Gynecology, and Pediatrics, University of Verona, University Hospital of Verona, Piazzale L.A. Scuro, 10, 37134 Verona, Italy

**Keywords:** pituitary neoplasms, pituitary disease, endoscopy, hypopituitarism, treatment outcome

## Abstract

Background: The endoscopic endonasal approach (EEA) is a well-established technique for the treatment of pituitary neuroendocrine tumor Preservation of normal gland tissue is crucial to retain effective neuroendocrine pituitary function. The aim of this paper is to analyze pituitary endocrine secretion after EEA for pituitary neuroendocrine tumor to identify potential predictors of functioning gland recovery. Methods: Patients who underwent an exclusive EEA for pituitary neuroendocrine tumors between October 2014 and November 2019 were reviewed. Patients were divided into groups according to postoperative pituitary function (Group 1, unchanged; group 2, recovering; group 3, worsening). Results: Among the 45 patients enrolled, 15 presented a silent tumor and showed no hormonal impairment, and 30 patients presented pituitary dysfunction. A total of 19 patients (42.2%) were included in group 1, 12 (26.7%) patients showed pituitary function recovery after surgery (group 2), and 14 patients (31.1%) exhibited the onset of new pituitary deficiency postoperatively (group 3). Younger patients and those with functioning tumor were more likely to have complete pituitary hormonal recovery (*p* = 0.0297 and *p* = 0.007, respectively). No predictors of functional gland worsening were identified. Conclusion: EEA for pituitary neuroendocrine tumor is a reliable and safe technique regarding postoperative hormonal function. Preserving pituitary function after tumor resection should be a primary goal in a minimally invasive approach.

## 1. Introduction

In recent years, the endoscopic endonasal approach (EEA) has become a well-established and safe technique for the treatment of pituitary neuroendocrine tumor (PitNET) [1,2,3,4,5,6]. The primary goal of this kind of surgery is the decompression of neurovascular structures surrounding the sellar space, such as the cerebral trunk or optic chiasm; therefore, many studies have been published evaluating factors affecting the extent of resection and clinical recovery from symptoms such as headache and disturbance of visual and olfactory function [7,8,9]. Concurrent with tumor removal, preservation of normal gland tissue is crucial to provide an effective neuroendocrine pituitary function after surgery, thus avoiding the need for supplementary hormonal therapy. Investigation of pituitary secretion is therefore mandatory to correctly assess the effects of surgery and yet few reports have been published on this topic [10,11,12,13].

The aim of this preliminary report is to analyze pituitary endocrine secretion after EEA for pitNET performed at our institution in order to identify potential predictors of functional gland recovery or worsening.

## 2. Materials and Methods

A retrospective chart review was conducted on patients who underwent an EEA for treatment of pitNET between October 2014 and November 2019 at our Referral Skull Base Center. Inclusion criteria were: (1) exclusive EEA approach to the tumor, (2) tumor diameter > 1 cm in any plane, (3) postoperative histologically confirmed diagnosis of pitNET. Pituitary microadenomas were excluded because their small size prevents a mass effect on the surrounding normal gland tissue; therefore, in these cases, preoperative and postoperative hormonal impairment related to mass effect and surgical maneuvers, respectively, are generally not observed.

Patients underwent a preoperative and postoperative (3 months after surgery) dedicated magnetic resonance imaging (MRI) pituitary protocol. Radiological characteristics were evaluated first by one of the authors and then validated by a second observer with emphasis on T1 contrast enhanced and T2 sequences in axial, coronal, and sagittal images. Tumor size was assessed by measuring its major axis in any plane. Cavernous sinus invasion was graded according to the modified Knosp score [14,15] and this grading was confirmed in all cases by surgical evidence, intraoperatively.

The extent of resection was classified, based on the 3-month postoperative MRI, as follows: (1) Gross total resection (GTR), when there was absence of residual tumoral tissue, (2) Near-total resection (NTR), in cases showing a small tumoral residual, recognized in at least two consecutive MRI slices and in two different planes, close to neurovascular structures (optic chiasm, healthy pituitary gland, internal carotid artery), despite the fact that a complete resection had been planned, (3) Subtotal resection (STR), when only a debulking was preoperatively planned for a giant invasive pituitary tumor. In these patients, the main goal was decompression of neurovascular structures to restore or prevent worsening of neurological symptoms.

Laboratory tests were used to define hormonal pituitary assessment; pituitary function was evaluated preoperatively and 6 months postoperatively in all patients. No dynamic measurements were performed. Thyroid gland-related hypothyroidism was not contemplated as a defect in this study. 

Endocrine evaluation included five adeno–pituitary axes: adrenocorticotropic hormone (ACTH, reference values between 1.80 and 13.20 pmol/L) and cortisol (reference values between 133 and 537 nmol/L); thyroid-stimulating hormone (TSH, reference values between 0.30 and 4.20 mUI/L) and free T4 (reference values between 11.0 and 22.0 pmol/L); growth hormone (GH, reference value lower than 7.00 microg/L) and insulin-like growth factor 1 (IGF-1, reference values between 8.00 and 26.00 nmol/L); prolactin (PRL, reference values between 102 and 496 mIU/L); follicle-stimulating hormone (FSH, reference value according to the ovarian cycle), and luteinizing hormone (LH, reference value according to ovarian cycle), and, depending on patient sex, estradiol and/or free and total testosterone. To assess ACTH deficiency and the presence of ACTH secerning tumor, ACTH and cortisol blood levels were analyzed [16]. To determine GH deficiency, the measurements of IGF-1 were considered. Low serum IGF-I levels in patients with ≥3 additional pituitary hormone deficiencies after pituitary surgery diagnosed GH deficiency in the absence of GH stimulation testing [17,18].

To evaluate posterior pituitary gland function, urine osmolarity was checked. The diagnosis of postoperative insipidus diabetes was based on polyuria with low urine osmolarity [19]. 

Data were collected in a Microsoft Excel (Microsoft Corp., Redmond, WA, USA) spreadsheet and updated periodically.

Patients were divided into three groups based on their postoperative pituitary function compared to the preoperative function, as follows. Unchanging group (group 1): patients showing unchanged pituitary function after surgery. Recovering group (group 2): patients with a postoperative improvement in pituitary function. Worsening group (group 3): patients with postoperative worsening of pituitary function.

We decided to evaluate the factors which could be potential predictors of gland recovery or deficiency after surgery. We analyzed the impact of sex, age, maximum tumor diameter, Knosp grade, presence of tumoral residual, presence of functioning tumor, previous surgery, and intraoperative cerebrospinal fluid (CSF) leakage.

Statistical analysis was performed by Fisher’s exact test and Student’s *t*-test to assess differences between groups. Statistical significance was assessed at the level α = 0.05. The normality of data distribution was assessed with the Kolmogorov-Smirnov test. The assessment of the normality of data distribution was performed as a prerequisite for Fisher’s exact test and Student’s *t*-test.

## 3. Results

Among a total of 47 patients who underwent an EEA for pitNET at our Referral Skull Base Center in the period examined, based on inclusion criteria, 45 were admitted to this study.

### 3.1. Patient Demographics

Out of 45 patients enrolled, 26 were male (57.7%), and 19 were female (42.3%). Age at time of surgery ranged from 21 to 79 years (mean age 56.9, SD 14.5).

### 3.2. Radiological Characteristics: Tumor Size and Knosp Grade

Preoperative MRI demonstrated a mean maximum tumor diameter of 28.13 mm (range, 12–79 mm, SD 14.24).

Knosp grade was 0 in 9 patients (20%), 1 in 12 patients (26.7%), 2 in 12 patients (26.7%), 3 in 1 patient (2.2%) and 4 in 11 patients (24.4%). Cavernous sinus invasion (Knosp grade 3 and 4) was observed radiographically in 12 patients (26.7%).

### 3.3. Extent of Resection

A standard fully endoscopic transsphenoidal transsellar approach was conducted in most cases (73.3%, 33 patients). The remaining 12 patients (26.7%) underwent an expanded transsellar-trans-planum approach. GTR was achieved in 33 (73.3%) patients, NTR in 10 (22.2%) patients and STR in 2 (4.4%) patients. Suprasellar cistern invasion was seen in 16 patients (35.5%). Therefore, the presence of residual tumoral tissue was observed overall in 12 (26.7%) patients.

### 3.4. History of Previous Pituitary Surgery

A total of eight patients (17.8%) had presented with recurrent tumors after previous transsphenoidal surgery at another hospital center.

### 3.5. Preoperative Pituitary Function

Among the 45 patients, 15 presented with a silent tumor and showed no hormonal impairment, while 30 patients presented with preoperative pituitary dysfunction: 16 patients presented a functioning tumor and 14 patients presented a silent tumor with a deficit disorder in at least 1 hormonal release.

Among the functioning tumor patients (16), 7/16 presented growth hormone (GH) secreting tumors, 7/16 medically resistant prolactinomas (PRL), 1/16 thyroid-stimulating hormone (TSH) and 1/16 adrenocorticotrophic hormone (ACTH) secreting tumor. Four out of 16 patients also had hypofunctional pituitary changes, with deficit disorders in at least 1 hormone.

Among the silent tumor patients (29), 15/29 presented no hormonal impairment and 14/29 a deficit disorder, 8/14 presented hypogonadism, 8/14 hypothyroidism, 6/14 hypoadrenalism, and 3/14 GH-deficit. PRL was oversecreted in 3/14 patients (due to pituitary stalk compression) and under-secreted in 3/14 patients. No patient presented diabetes insipidus.

### 3.6. Postoperative Pituitary Function

Overall, postoperative pituitary function in our series was unchanged or improved in 21 patients (46.7%), whereas in 24 patients (53.3%) a new hormonal deficiency was observed in at least one hormonal axis. The mean functional deficiency was 2.7 hormones, with the loss of at least three hormones in 8 (17.8%) patients. Considering the type of hormonal imbalance after surgery, we reported 17 patients (37.7%) with hypoadrenalism, 11 patients (24.4%) with hypogonadism, 19 patients (42.2%) with hypothyroidism, three patients (6.6%) with GH deficit, and three patients (6.6%) with PRL deficit. Finally, two patients (4.4%) developed persistent diabetes insipidus, treated continuously with desmopressin.

Among the 16 patients with functioning tumor, eight cases showed a normalization of pituitary function after surgery. Eight patients presented a deficiency in at least one hormonal axis: in three patients the hormonal deficiency was also present before surgery, while five patients developed a new deficit in one or more hormonal release after the transsphenoidal transsellar approach. Out of the last wight patients with pituitary deficiency, four patients showed the persistence of hormonal hypersecretion observed preoperatively (one GH and three PRL secreting tumor).

Among the 29 patients affected by a silent tumor, nine patients showed a new deficit in at least in one hormonal axis postoperatively. Nine patients with normal preoperative pituitary function did not show any deficit after surgery, seven patients with a preoperatively deficit in at least one hormonal release maintained the same deficiency after surgery, while four patients, with at least one preoperative hormonal deficiency, exhibited a completely pituitary gland recovery after EEA. Further details regarding all patients’ hormonal results are reported in the Supplementary Materials. 

Patients were sub-classified into three groups according to hormonal secretion detected preoperatively and 6 months after surgery. 

A total of 19 patients (42.2%) were included in the unchanging group (group 1): in nine cases (20%), a normal preoperative pituitary function persisting after surgery was observed, whereas in 10 patients (22.2%), the hormonal deficiency detected before surgery remained unchanged postoperatively. The recovering group (group 2) consisted of 12 (26.7%) patients showing pituitary function normalization postoperatively. A total of 14 patients (31.1%) were included in the worsening group (group 3), exhibiting the onset of a new pituitary deficiency postoperatively.

### 3.7. Recovering Group Characteristics (Group 2)

The characteristics of the 12 patients who presented complete pituitary recovery after surgery in terms of demographics, secreting tumor, tumor size, and extent of resection are shown in Table 1.

### 3.8. Worsening Group Characteristics (Group 3)

Out of 14 patients, five (35.7%) presented a functioning tumor. The mean age was 59 years (S.D. ± 14.3). The mean maximum diameter of treated pitNET was 29.1 mm (S.D. ± 15.6 mm). Knosp grade was 0 in two patients (14.3%), 1 in two patients (14.3%), 2 in six patients (42.9%), 3 in one patient (7.1%) and 4 in three patients (21.4%). Eleven patients underwent GTR, in two patients we performed a near total resection and in one patient a debulking of the lesion. The mean hormone deficiency was 2.7 and the hormones involved were TSH in 12/14, ACTH in 11/14, and gonadotropin in 7/14. Impairment in GH/IGF-1 release was detected in three patients; prolactin deficiency was found in three patients. No diabetes insipidus was identified in this group. Among this group, two patients had undergone previous surgery. Intraoperative CSF leak was observed in four patients. The features of patients in group 3 are summarized in Table 2.

### 3.9. Predictors of Pituitary Function Recovery or Worsening

Sex, maximum diameter, Knosp grade, tumoral residual and intraoperative CSF leak were not predictors of gland recovery. Younger patients (*p* = 0.0297) and those with functioning tumor (*p* = 0.007) were more likely to have complete pituitary hormonal recovery, as shown in Table 3.

The results were not statistically significant for all the factors tested (*p* > 0.2 in all cases) with regard to pituitary gland worsening (Table 4).

### 3.10. Patients Affected by Silent and Functioning Tumor: Sub-Classification into the Three Groups According to Hormonal Secretion

Among the 29 patients affected by silent tumors, 16 (55.2%) presented an unchanged pituitary function after surgery (group 1), nine (31%) showed a postoperative new hormonal deficiency (group 3) and only four (13.8%) exhibited a completely pituitary gland recovery (group 2).

The 16 patients affected by functioning tumors demonstrated a complete normalization of the pituitary function postoperatively (group 2) in eight cases (50%); three (18.7%) patients were included in the unchanged group (group 1) and five (31.3%) patients developed a deficiency in at least one hormonal release (group 3) after EEA.

The features of each group according to sex, age at time of surgery, maximum diameter, Knosp grade and tumoral residual are displaced in Table 5. Because of the limited sample size, the comparison between groups did not provide statistically significant data.

## 4. Discussion

Since its first report in 1992, a fully endoscopic endonasal approach to sellar lesions has become increasingly common and is actually considered the first choice for surgical treatment of pitNET [20]. In fact, the EEA shows similar rates of GTR and perioperative mortality [21,22,23,24,25] as for classic craniofacial approaches and ensures a better quality of life [1,13,26,27]. The main goal of these approaches is to remove pathological tissue to reduce the mass effect on critical neurovascular structures in close relationship to sellar space, especially the optic chiasm, interpeduncular and prepontine cisterns and brainstem. On this basis, in the last decade, many reports have been published on the outcomes of this surgery, with special regard to the extent of resection and its relationship to recovery from symptoms, especially headache, and disturbance of visual and olfactory function [7,8]. In addition, another key point during surgical maneuvers is the visualization and preservation of the unaffected gland tissue encased or displaced by the pathological tissue. This step is crucial in providing an effective neuroendocrine pituitary function after surgery, thus avoiding the need for supplementary hormonal therapy. The analysis of hormonal secretion is therefore a necessary step to correctly assess the effects of this kind of surgery on pituitary function, and several reports have been published on this topic [10,11,12,13].

Among the 45 patients in this study, 14 (31.1%) displayed a postoperative loss in at least one hormone (worsening group, group 3), 12 patients (26.7%) with preoperative hormonal impairment showed complete recovery of hormonal secretion (recovering group, group 2), whereas in 19 patients (42.2%), no change in gland functioning was detected (unchanging group, group 1). A multicenter prospective study conducted by Little et al. (2019) [13] reported that 21.1% of patients (20/95) experienced recovery in at least one axis, whereas 9.7% of patients (14/145) had developed at least one new deficiency. Elshazly et al. [28] evaluated 55 patients with giant pituitary tumor (>4 cm in maximum diameter) who underwent surgery with an EEA. A new hormonal deficit occurred in eight patients, whereas recovery of one or more hormonal axis deficits occurred in six patients. In the study conducted by Do et al. [29] on recurrent pituitary tumor, 14.8% of patients (9/61) developed single or multiple new anterior pituitary deficits after first surgical treatment.

With regard to the onset of new postoperative hormonal deficiency, the rate of hormonal loss observed in our experience was higher than in the aforementioned reports. Despite a challenging comparison, due to the absence of standardized benchmarks, we analyzed the patients in the worsening group to clarify these data. In 42.8% of cases (6/14 patients), a supradiaphragmatic or para-sellar space invasion was observed. Although the relationship with suprasellar involvement and Knosp grade was not statistically significant, this result supports the idea that increasing tumor mass may lead to ischemic injury or direct destruction of healthy pituitary parenchyma, thus resulting in hormonal loss. This claim clearly needs to be verified by studies with a larger sample size, but nonetheless it is in agreement with the findings of other authors [27].

Regarding the type of hormonal deficiencies observed in the worsening group, in our study population the most common deficit reported was thyrotropin hormone (TSH), followed by adrenocortical hormone (ACTH) and gonadotropin (FSH/LH). This result is in contrast to other reports [30], where ACTH was the most frequently detected deficit after surgery, but at present there is insufficient data to clarify these differences.

An intriguing argument concerns the onset of postoperative diabetes insipidus, a rare complication of the EEA that is most frequently found in its transient form. Nayak et al. [31] revealed permanent diabetes insipidus onset in 4% of patients who underwent EEA. In their series of 271 patients, the presence of visual abnormalities, suprasellar extension, and maximal tumor diameter was significantly associated with an increased incidence of postoperative diabetes insipidus, both transient and permanent. In our series, no patient presented insipidus diabetes preoperatively. Because of the laboratory tests for hormonal pituitary assessment at 6 months postoperatively, transient diabetes insipidus was not evaluated in this study. Two patients (4.4%) developed persistent insipidus diabetes after surgery, treated continuously with desmopressin. Given the small sample size, it is not possible to perform a statistical analysis. Nonetheless, it is interesting to note that diabetes insipidus occurred in two patients with giant tumor with suprasellar involvement, and in which a CFS leak emerged during surgery. In our opinion, this finding suggests that extensive surgical manipulation, as in the event of a CSF leak, can lead to trauma to the gland or infundibulum tissue.

With regard to the recovering group, our findings are in accordance with those reported in several studies, in which recovery of preoperative hormonal deficits occurred in 10–30% of cases, varying from type to involvement of the lesions [12,28,30,32]. As a further observation, considering both the recovering and unchanging groups, in 68.9% of cases, no worsening of pituitary function was observed.

To better clarify the data analysis, we decided to focus our attention on selected potential predictors of postoperative function recovery or worsening. Considering tumor size, Fatemi et al. [10] demonstrated that, the larger the tumor, the greater the risk of pituitary gland failure; they indicated a size of 20 mm as the upper limit; beyond 20 mm, the pituitary failure rate is increased. In our cohort, among the 31 patients with tumor size > 20 mm, 10 experienced postoperative new hormonal loss (32.2%), whereas out of 14 patients with tumor size <20 mm, four (28.6%) showed a new deficiency. Even though this result was not statistically significant, we believe this finding is probably related to the greater surgical handling occurring in larger tumors. In addition, as previously reported by Nomikos et al. [27], it is interesting to observe that tumor size affected gonadotropin release more than other hormonal axes. In fact, we detected 11 cases with postoperative gonadal loss and these patients presented a mean maximum tumor diameter of 38.2 mm.

While surgery to an increasing size of tumor has a negative impact on the function of healthy gland tissue postoperatively, removal of that tumor mass may lead to a greater improvement in hormonal release, due to the mass decompression effect. In this regard, the main regularized hormone after surgery in our series was prolactin, followed by stabilization of gonadotropin and adrenocortical hormone deficiency. Previous studies on this topic [27,33] observed that preoperative hyperprolactinemia typically results from compression of the pituitary stalk, and decompression maneuvers mainly affect prolactin release compared with other hormones; thus, prolactin blood level could be considered a useful predictor of postoperative recovery of pituitary function [27,33]. Infundibular compression is the main mechanism, affecting the delivery of hypothalamic hormones and determining hypopituitarism, thus explaining the better recovery rate in these patients.

Regarding the role of functioning tumor in conditioning postoperative gland function, a few studies have evaluated the impact of the EEA on enhanced hormonal secretion. In a series of 142 prolactinomas, Akin et al. [34] showed that 74.6% of patients went into remission after the EEA. Concerning GH-secreting pitNET, endocrinological cure was achieved in 46–61% of patients after the EEA [35,36,37,38]. The presence of a functioning tumor was a strong predictor of postoperative gland recovery (*p* = 0.007). According to the authors, this result was not related to tumor size, but to the earlier diagnosis in functioning compared to silent tumors.

Furthermore, in agreement with the report by Webb and colleagues [30], the rate of complete hypopituitarism recovery in patients with GH releasing tumor was greater than in the other patients (4/7); these patients were typically younger, and both the hormonal therapy before surgery and their high IGF-1 levels helped to preserve pituitary gland activity.

As reported by other authors [10,27], in our cohort, age was a significant predictor of pituitary restoration (*p* = 0.0297): younger patients presented a better pituitary function after endoscopic surgery compared to the others, despite preoperative pituitary gland status or tumor size.

The last noteworthy consideration concerns the role of the intraoperative CSF leak. Fatemi et al. [10] observed that this is related to a worse hormonal postoperative function and reflects the more extensive surgical manipulation of the infundibulum and gland. Although, in our series, the statistical analysis did not reveal a significant result, among nine patients who experienced intraoperative CSF leak, only one presented postoperative pituitary gland recovery. Furthermore, this finding could be predictive of permanent diabetes insipidus [39]. In fact, both patients who developed persistent diabetes insipidus showed a dural defect with an intraoperative CSF leak. 

This study may help to establish standardized benchmarks in the evaluation of functional pituitary outcome after endoscopic approaches to pitNET. 

The absence of hormones’ dynamic measurements and the low sample size are the main limitations of this study, and these preliminary results need to be validated in studies with a larger sample size.

## 5. Conclusions

This preliminary report confirms that EEA for pitNET is a reliable technique with regard to postoperative hormonal function. This is supported by the finding that only a minority of patients needed replacement hormonal therapy after surgery. The young age and the presence of functioning tumor proved to be predictors of functional gland recovery after surgery. No predictors of functional gland worsening were identified in our cohort. Nonetheless, the increasing size of the tumor and the presence of intraoperative CSF leak may play a role in the development of new postoperative hormonal loss. This finding could be related to more extensive surgical manipulation, as already reported in other experiences. Preserving pituitary function after pitNET resection is crucial for patients’ hormonal balance and this should be a primary goal in a minimally invasive approach.

## Figures and Tables

**Table 1:** Features’ description for patients who showed a pituitary gland recovery after endoscopic transsphenoidal surgery (group 2).

#	Age	Sex	Maximum Diameters (mm)	KNOSP Grade	Resection	Functioning pitNET	Prior Surgery	Intraoperative CSF Leak	Type of Impairment after Surgery
1	53	M	15	1	GTR	Yes (GH)	No	No	-
2	43	M	19	0	GTR	No	No	No	-
3	43	F	20	0	NTR	No	No	No	-
4	47	M	25	2	GTR	No	No	No	-
5	33	M	51	4	NTR	Yes (TSH)	No	No	-
6	50	F	18	0	GTR	Yes (ACTH)	No	No	-
7	58	F	42	4	GTR	Yes (PRL)	No	No	-
8	30	F	18	1	GTR	No	No	No	-
9	69	M	12	1	GTR	Yes (GH)	No	No	-
10	72	F	13	1	GTR	Yes (GH)	No	No	-
11	64	F	12	0	GTR	Yes (GH)	No	No	-
12	25	F	21	1	GTR	Yes (PRL)	No	Yes	-

**Table 2:** Features’ description for patients who exhibited the onset of new pituitary deficiency after endoscopic transsphenoidal surgery (group 3). Legend: ↓: under the reference value; ↑: over the reference value.

#	Age	Sex	Maximum Diameters (mm)	KNOSP Grade	Resection	Functioning pitNET	Prior Surgery	Intraoperative CSF Leak	Type of Impairment after Surgery
1	21	M	21	0	GTR	Yes (PRL)	No	No	TSH, LH/FSH ↓ PRL ↑
2	60	M	58	2	NTR	No	No	Yes	ACTH, TSH, LH/FSH ↓
3	52	M	12	0	GTR	Yes (GH)	No	No	TSH, PRL ↓ GH/IGF-1 ↑
4	49	M	32	2	GTR	No	No	No	ACTH, TSH, LH/FSH, PRL ↓
5	65	M	62	3	NTR	No	Yes	Yes	ACTH, TSH, LH/FSH and GH/IGF-1 ↓
6	67	F	17	2	GTR	No	Yes	No	TSH ↓
7	64	F	22	2	GTR	Yes (PRL)	No	No	ACTH, TSH ↓
8	53	M	15	1	GTR	Yes (GH)	No	No	ACTH, TSH, LH/FSH, PRL ↓
9	55	M	24	2	GTR	No	No	No	ACTH, TSH, LH/FSH, GH/IGF-1 ↓
10	77	M	25	4	GTR	No	No	No	ACTH ↓
11	51	M	15	2	GTR	Yes (GH)	No	No	ACTH, TSH ↓
12	66	M	31	1	GTR	No	No	No	ACTH ↓
13	67	F	28	4	GTR	No	No	Yes	ACTH, TSH ↓
14	79	M	45	4	STR	No	No	Yes	ACTH, TSH, LH/FSH, GH/IGF-1 ↓

**Table 3:** Predictors of gland recovery following transsphenoidal surgery. *p* values < 0.05 are shown in bold.

Predictor	Group 1	Group 2	*p* Value
N. patients	19	12	
Age in years, mean (±SD)	60.5 (±12.7)	48.9 (±15.1)	0.0297
Male sex, number (%)	11 (57.9%)	4 (33.3%)	0.2734
Maximum diameter, mm mean (±SD)	31.2 (±13.9)	22 (±12.1)	0.0753
KNOSP grade			
0–2	13	10	0.4325
3–4	6	2	
Tumoral residual	7	2	0.4184
Functioning pitNET	3	8	0.007
Prior surgery	6	0	0.0585
Intraoperative CSF leak	4	1	0.6236

**Table 4:** Predictors of new pituitary gland deficiency following transsphenoidal surgery.

Predictor	Group 1	Group 3	*p* Value
N. patients	19	14	
Age in years, mean (±SD)	60.5 (±12.7)	59 (±14.3)	0.7577
Male sex, n (%)	11 (57.9%)	11 (78.6%)	0.2783
Maximum diameter, mm mean (±SD)	31.2 (±13.9)	29.1 (±15.6)	0.6819
KNOSP grade			
0–2	13	10	1
3–4	6	4	
Tumoral residual	7	3	0.4551
Functioning pitNET	3	5	0.2379
Prior surgery	6	2	0.4157
Intraoperative CSF leak	4	4	0.6951

**Table 5:** Features’ description for silent tumors and functioning tumors’ patients subclassified into three groups according to postoperative pituitary gland function.

Silent Tumors (29 Patients)			
Variables	Group 1	Group 2	Group 3
N. patients	16	4	9
Age in years, mean (± SD)	64.5 (±9.1)	40.75 (±7.41)	65 (±9.55)
Male sex, number (%)	9 (56.25%)	2 (50%)	7 (77.8%)
Maximum diameter, mm mean (± SD)	31.81 (±15.17)	20.5 (±3.10)	35.8 (±15.69)
KNOSP grade			
0−2	12	4	5
3−4	4	0	4
Tumoral residual	6	1	3
Functioning Tumors (16 Patients)			
Variables	Group 1	Group 2	Group 3
N. patients	3	8	5
Age in years, mean (±SD)	39 (±3.60)	53 (±16.71)	48.2 (±16.08)
Male sex, number (%)	2 (66.7%)	2 (25%)	4 (80%)
Maximum diameter, mm mean (±SD)	28 (±1.73)	23 (±15.02)	17 (±4.30)
KNOSP grade			
0−2	1	6	5
3−4	2	2	0
Tumoral residual	1	1	0

## Data Availability

Full data are available from the corresponding author upon request.

## Supplementary Materials

The following supporting information can be downloaded at: https://www.mdpi.com/article/10.3390/jcm12082986/s1.
